# Automatic kernel counting on maize ear using RGB images

**DOI:** 10.1186/s13007-020-00619-z

**Published:** 2020-06-01

**Authors:** Di Wu, Zhen Cai, Jiwan Han, Huawei Qin

**Affiliations:** 1 Institute of Mechanical Engineering, Hangzhou Dianzi University, Hangzhou, 310018 Zhejiang People’s Republic of China; 2 School of Software, Shanxi Agricultural University, Taigu, 030801 Shanxi People’s Republic of China; 3 Ocean College, Zhejiang University, Zhoushan, 316021 Zhejiang People’s Republic of China

**Keywords:** Kernel recognition, Counting, Computer vision, Adaptive threshold, Local Maxima

## Abstract

**Background:**

The number of kernels per ear is one of the major agronomic yield indicators for maize. Manual assessment of kernel traits can be time consuming and laborious. Moreover, manually acquired data can be influenced by subjective bias of the observer. Existing methods for counting of kernel number are often unstable and costly. Machine vision technology allows objective extraction of features from image sensor data, offering high-throughput and low-cost advantages.

**Results:**

Here, we propose an automatic kernel recognition method which has been applied to count the kernel number based on digital colour photos of the maize ears. Images were acquired under both LED diffuse (indoors) and natural light (outdoor) conditions. Field trials were carried out at two sites in China using 8 maize varieties. This method comprises five steps: (1) a Gaussian Pyramid for image compression to improve the processing efficiency, (2) separating the maize fruit from the background by Mean Shift Filtering algorithm, (3) a Colour Deconvolution (CD) algorithm to enhance the kernel edges, (4) segmentation of kernel zones using a local adaptive threshold, (5) an improved Find-Local-Maxima to recognize the local grayscale peaks and determine the maize kernel number within the image. The results showed good agreement (> 93%) in terms of accuracy and precision between ground truth (manual counting) and the image-based counting.

**Conclusions:**

The proposed algorithm has robust and superior performance in maize ear kernel counting under various illumination conditions. In addition, the approach is highly-efficient and low-cost. The performance of this method makes it applicable and satisfactory for real-world breeding programs.

## Background

Maize is one of the most important crops in the world [1]. Kernel-trait scoring is an important part of the maize breeding process, where the number of kernels per ear is a key indicator for maize quality assessment [2]. Traditional kernel counting methods rely on simple observation by humans [3]. These methods are labour-intensive, time-consuming and low-efficient, often leading to errors. The photocell technology is developed to automatically count maize kernels, but a photocell whose lifetime is extremely short and sensitivity decreases with running time needs to be replaced frequently [4].

Machine vision enabled systems can acquire phenotypic information in a high-throughput manner [5]. This type of technology is being used increasingly in the extraction of trait information from cereals [6–9] including maize. Information about cereal ears and the seeds/kernels in the ears can be acquired from images using one of two main methods. The first method involves rotating ears and acquiring images to obtain full-surface image information [10]. However, rotatory mechanism increases the manufacturing cost of the entire system and decreases working efficiency or throughput. An alternative method takes a single image of the ear, extracts features and estimates the total number of kernels [11]. The quality of estimation depends on several factors but this method is much cheaper and could be adapted to suit breeding programs. In 2009, Ruiz et al. modified the EASA algorithm for environmentally adaptive crop segmentation [12].

Images invariably show complex arrangements of kernels and Zhao et al. used a median filter and a Wallis filter as a pre-processing method. It is followed by an improved Otsu method with a combination of multi-threshold and row-by-row gradient based method (RBGM) gradient descent to separate the kernels [13]. In 2018, Zhang et al. proposed a segmentation method for kernels on maize ear, which combines a genetic algorithm with improved pulse coupled neural network. It achieved an accuracy of 98% [14]. However, touching kernels were not considered and influenced the overall performance. Accurate counting requires identification and separation of touching kernels. In 2017, Grift et al. proposed a semi-automated vision system to count the number of kernels in the mid-section of a maize ear and to calculate a range of morphological parameters of the kernel. They used an area threshold to distinguish touching kernels, which were then separated by a local Otsu threshold [15]. The number of kernels on the base and tip of the ear was estimated by a formula with errors ranging from − 7.67 to + 8.60%.

Liu et al. improved the watershed algorithm with morphological multi-scale decomposition to separate rice kernels [16]. Subsequently, Belan et al. developed a marker-controlled watershed algorithm based on the kernels constrained by path-cost function and distance threshold in Euclidean Distance Transform (EDT) with an 86.2% accuracy rate [17].

In 2001, Visen et al. proposed a method to distinguish between a group of touching kernels and an isolated kernel by the degree of overlap between each kernel and its equivalent ellipse. Then a second method separated the touching kernels by evaluating the boundary curvature to determine the open nodes and touching line [18]. In 2011, Mebatsion et al. improved the segmentation algorithm based on the concavity with the elliptic Fourier series approximation smoothing the boundary contours [19]. Plot detection algorithms based on a background skeleton were also used to separate the touching kernels [20]. Active Contour Model (Yang et al. 2010) and Morphological operations (Wang et al. 2006 and Porto et al. 2008) were also popular in separation of the touching kernels [21–23].

Previous work demonstrated reasonable performance. However, the colour gradient between kernels in images of maize ear is often narrow and it is difficult to segment kernels using colour information only. There are three simultaneous problems: corner-to-corner, edge-to-corner and edge-to-edge touching. Also, both the sizes and the shapes of kernels on the same maize ear are irregular. These issues were not discussed in depth in previous publication.

The watershed and its improved versions are often accompanied with over-segmentation, that multiple false positive internal markers were detected in the same area. The concavity algorithm, despite excellent improvements, could only separate a maximum of three touching kernels [18]. The combined ellipse-fitting and concavity algorithm was restricted to the separation of approximately elliptical kernels [19], which was not always suitable for maize kernels. The morphological operation and Active Contour Model failed to correctly segment the edge-to-edge touching and occluded kernels, and the latter was especially time-consuming.

Assessment of other objects pose similar problems and many algorithms have been developed for segmentation/recognition of fruits [24–28], cells/nuclei [29–32], fungal spores [33], disease spots [34] and defects [35]. However, these algorithms are not developed for the separation and recognition of kernels in images of maize ears, therefore, often problematic. Hence, it is necessary to develop an effective method for recognition and quantification of maize kernels in the ear images.

## Methods

### Plant material

Some maize samples are collected at a farmhouse in Suzhou City, Anhui Province. Other sample images were provided by DongYang Maize Research Institute of Zhejiang Academy of Agricultural Sciences. The test samples include eight varieties Zhengdan 958, Xianyu 688, Fudan No. 3, Xianyu 335, Jinnuo 685, Jingkenuo 2016, Beibainuo No. 10, and Zhengbai No. 1. The sample images were taken from one side of the maize ears. The ground truth for testing the algorithm is acquired by manually counting kernel number in images. A total of 2000 maize ears were used in the algorithm development, of which 1200 ears were used for algorithm validation.

### Image acquisition

**Fig. 1:**
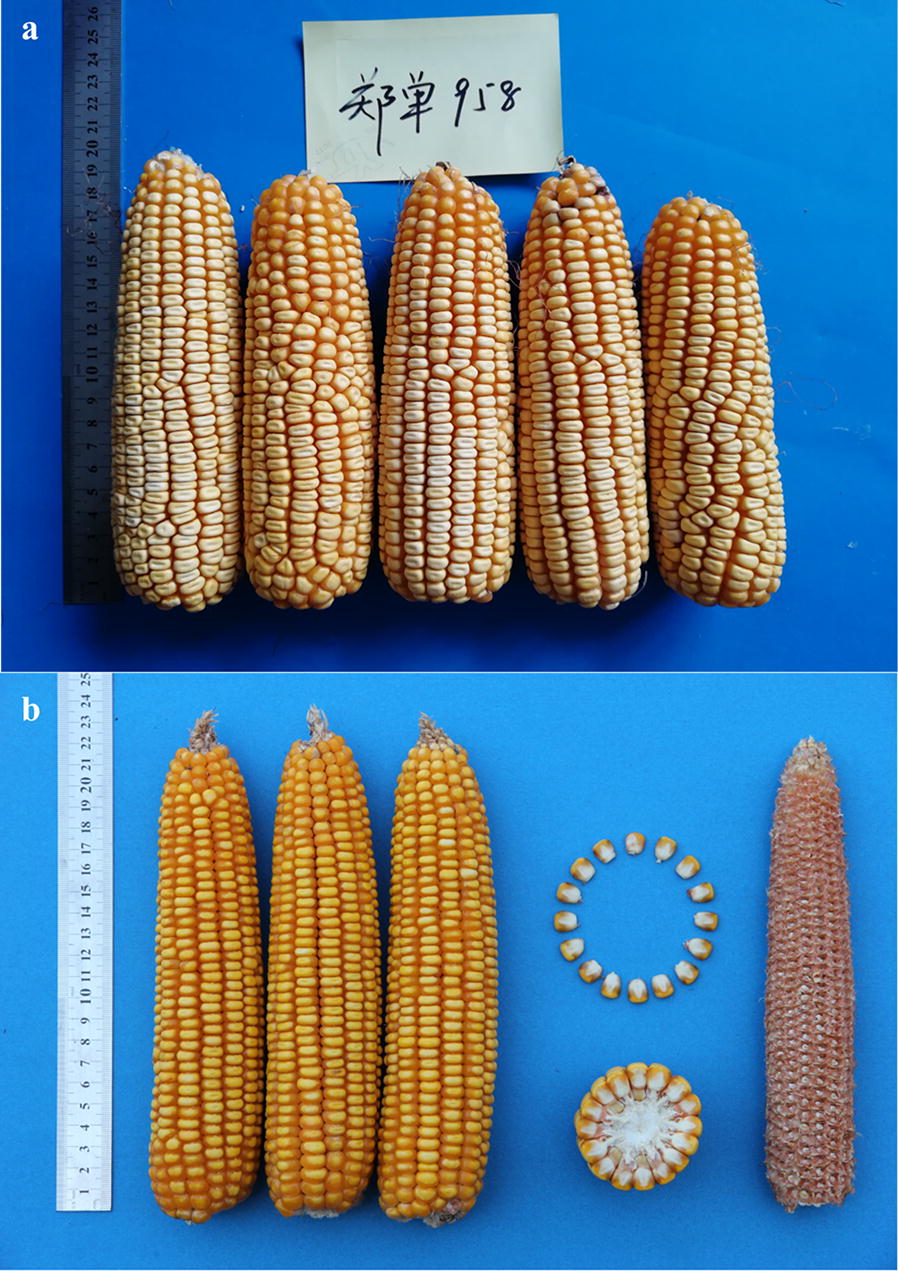
Maize ear images acquired under different lighting conditions: **a** under indoor illumination, **b** under outdoor illumination

Image quality is sensitive to the illumination as shown in Fig. 1. Images were acquired separately under indoor LED diffuse and outdoor natural diffuse illumination. Sample images of multiple maize varieties taken under various illuminations were selected to develop and test the proposed algorithm. Images were taken using a Canon camera (Cyber-shot EOS550D, 18 megapixels) and are 24-bit (RGB) colour images. The background is in blue as maize ears rarely contain blue colour. This can ease the segmentation of maize ear. The kernel recognition software was developed using Microsoft Visual Studio 2013(C++) with the following computer configuration: Intel(R) Core (TM) i5-4210U CPU @ 1.70 GHz 2.40 GHz, 12G (RAM).

### Image processing

#### Image compression

A reasonable compression of the image size, such as the Gaussian Pyramid [36], often helps improve the efficiency of extracting useful information. The advantages of Gaussian Pyramid are not only removing colour and pixel redundancy but also preserving the images’ low pass information. Level i $G_{i} \left( {x,y} \right)$ in Gaussian pyramid is calculated according to Eq. (1), where $w\left( {m,n} \right)$ is the kernel window function and $G_{i - 1} \left( {x, y} \right)$ is the compressed pixel value in level i-1.

1 $$ G_{i} \left( {x,y} \right) = \mathop \sum \limits_{m = - 2}^{2} \mathop \sum \limits_{n = - 2}^{2} w\left( {m,n} \right)G_{i - 1} \left( {2x + m,2y + n} \right) $$

**Fig. 2:**
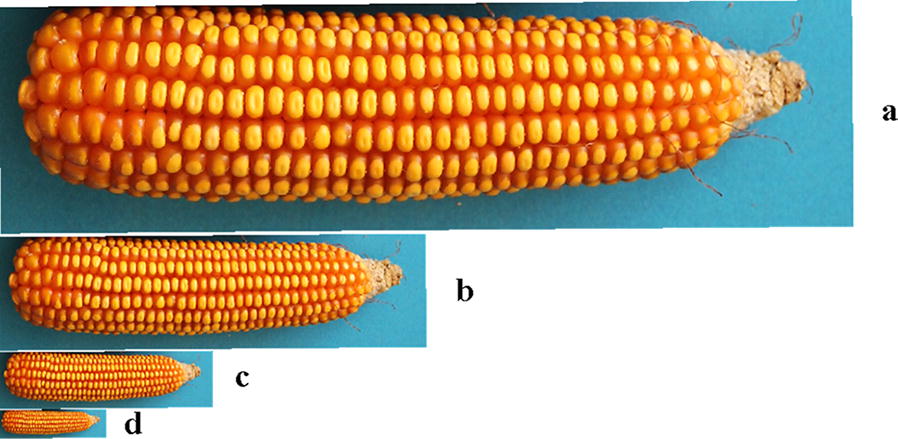
Compression layers in maize image Gaussian pyramid: **a** G_0_ layer (original image), **b** G_1_ layer, **c** G_2_ layer, **d** G_3_ layer

Figure 2 shows G_0_-G_3_ layers in maize image Gaussian pyramid according to the same ratio.

**Fig. 3:**
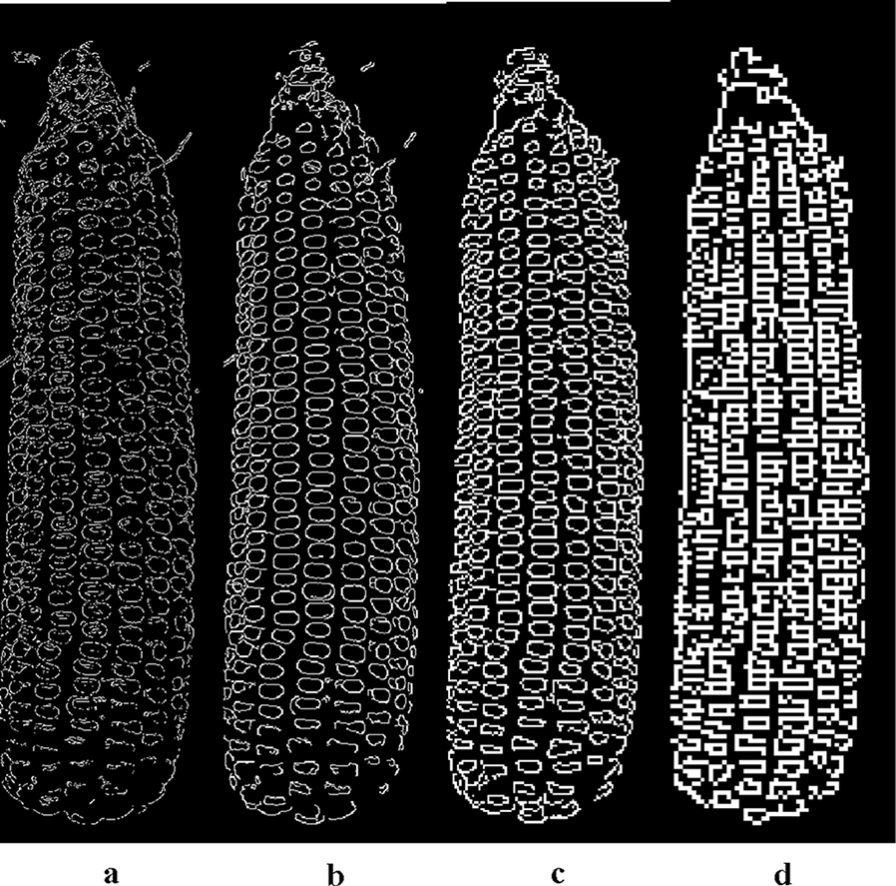
Canny edge detection: **a**–**d** are edge maps of G_0_, G_1_, G_2_, G_3_, respectively

We first analyzed compressed image based on edge integrity and compression ratio. Canny operator [37] was used to detect edges of layer images G_0_-G_3_ as shown in Fig. 3, whose hysteresis thresholds are uniformly set to 80 and 80*2.2. These thresholds were established by testing a range of values and these gave the best results. For comparison purposes, the edge detection results are displayed for each kernel at the same scale in Fig. 3. Both G_1_ and G_2_ maintain relatively complete edge information (Figs. 3b, c). Considerable edge information in layer G_3_ was missing (Fig. 3d), which has a detrimental effect on the later segmentation.

The compression ratio R is calculated according to Eq. (2), where B is the bit number of the original image, B’ is the bit number of the compressed image.

2 $$ R = B '/B \times 100{{\% }} $$

Processing speed is inversely proportional to R. The processing speed gradually increased from G_0_ to G_3_ layer as R is reduced. Considering the above two factors, layer G_2_ is considered to be better for the balance of speed and quality. The compressed image was then restored in a Gaussian Pyramid [38]. Level *i*$G_{i} \left( {x,y} \right)$ in a Gaussian Pyramid is calculated according to Eq. (3), where $w\left( {m,n} \right)$ is the kernel window function and $G_{i - 1}$ is the pixel value in level *i*-*1*.

3 $$ G_{i} \left( {x,y} \right) = 4\mathop \sum \limits_{m = - 2}^{2} \mathop \sum \limits_{n = - 2}^{2} w\left( {m,n} \right)G_{i - 1} \left( {\frac{x + m}{2},\frac{y + n}{2}} \right) $$

#### Background separation

The first step of kernel recognition is to remove the background and the bald tip area, so that subsequent processing can be focused on maize kernel area. A threshold segmentation [39] method based on colour feature is adequate in the case as there is a clear boundary in colour intensities between the maize kernel and the to-be-removed (background and bald tip area) area.

### Mean shift filtering

Mean Shift Filtering [40] algorithm is a general clustering algorithm that replaces the original pixel value with the pixel value of the convergence point iteratively. This removes the local similar texture and retains the features with large differences such as edge, which makes it suitable to group kernel pixels with similar colours.

Each pixel in the image is a sample point. In this process, it is crucial to set the parameters, physical space radius *sp* and the colour space radius *sr*, for iteration space centered on the sample point. The smaller *sp* and *sr*, the more details remain; the larger *sp* and *sr*, the smoother the image. *sp* and *sr* are set to 40 and 60 respectively based on tests for the balance of kernel smoothness and fruit edge. Maximum layer numbers of the pyramid is set to 3.

**Fig. 4:**
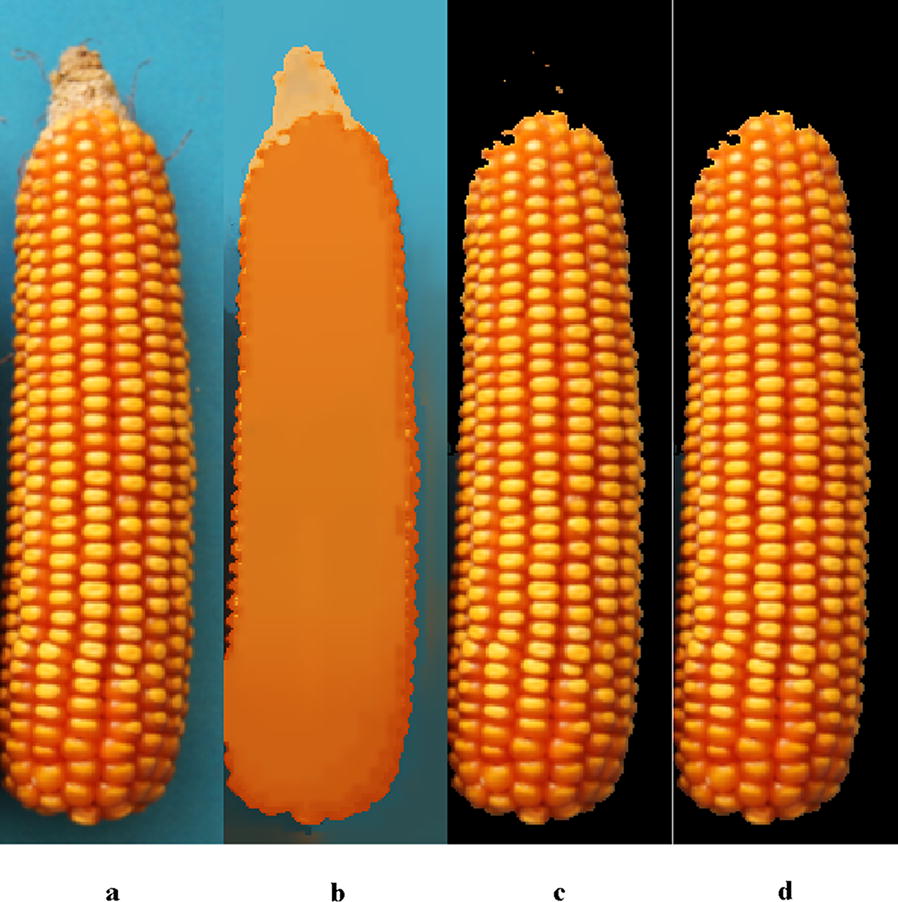
Result images of maize after the Mean Shift Filtering algorithm and threshold segmentation: **a** compressed maize image; **b** result image after the Mean Shift Filtering algorithm; **c** fruit image after threshold segmentation; **d** fruit image after noise removal

**Fig. 5:**
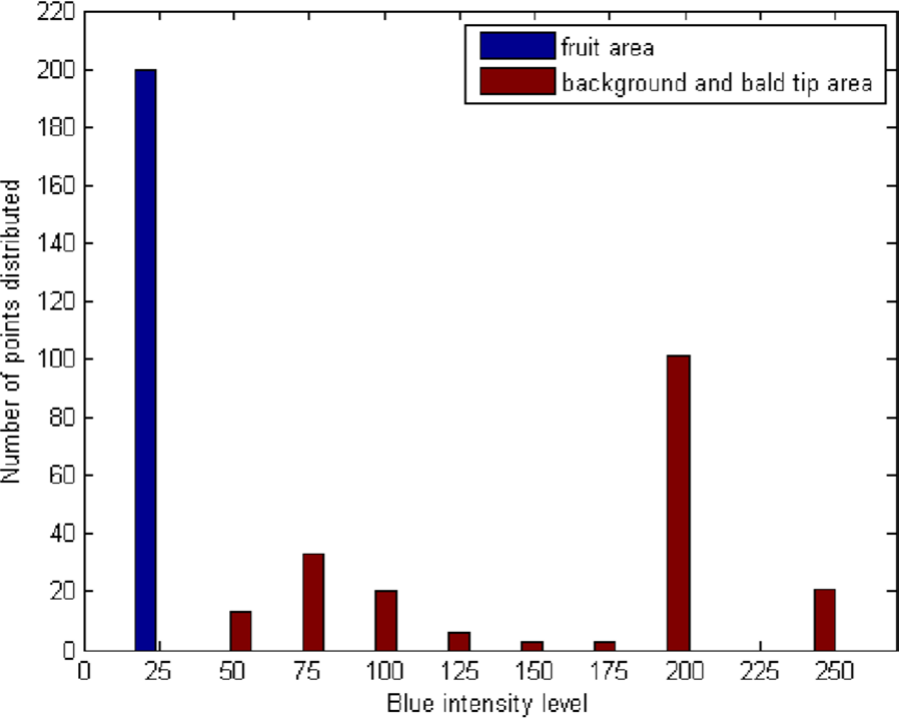
Statistical analysis of the filtered image Blue component

Figure 4a is the compressed image in previous step. Figure 4b is the result image after Mean Shift Filtering. Figure 5 shows the maize fruit area and to-be-removed area, taking blue as the segmentation colour feature (due to background is blue) and showing the observable differences. The maize fruit is segmented out by setting the unified threshold within blue channel, as shown in Fig. 4c. There was some noise in the processed image after segmentation. To remove noise, firstly, the operation of region filling was used to fill the holes in the kernels; Secondly, areas less than 20 pixels were removed using small-area removal. The result was shown in Fig. 4d.

#### Enhancement of the kernel edges

The touching area between kernels was often fuzzy and the colour gradient of this area was narrow. This could result in errors in subsequent kernel segmentation as the segmentation of kernels was mainly based on the clear definition of kernels edges. Here, colour deconvolution (CD) algorithm [41] was used to widen colour gradients in touching area to enhance the kernel edge.

### Colour deconvolution

In 2001, CD algorithm was initially proposed for separation and quantification of immunohistochemical staining [41]. Here, we make an assumption that the colour of maize fruit is made up of two or three “stains” (in this case, a new colour space). Kernel edge enhancement can be viewed as a problem of finding a stain with a major colour difference in touching areas. The amount distribution of an individual stain in maize fruit image is calculated by CD algorithm.

**Fig. 6:**
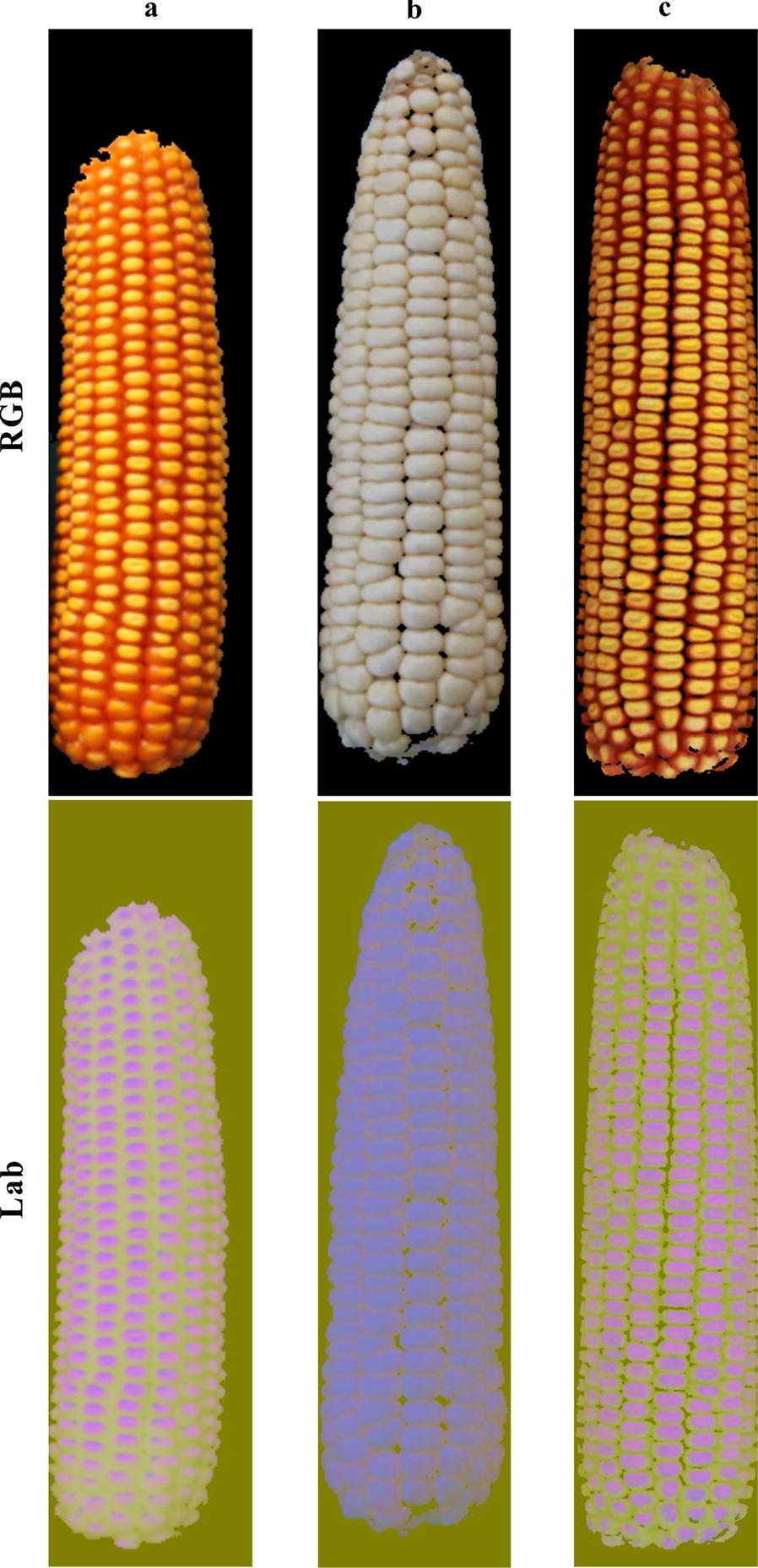
Maize fruit images in RGB colour space and converted Lab colour space, the varieties are in turn: **a** Xianyu 688; **b** Jinnuo 685; **c** Fudan No. 3

Firstly, the colour space was transformed from the RGB colour space to the Lab colour space which has a wider colour range [24], according to Eq. (4). Hue and brightness are two separate channels in Lab space, which was considered an advantage over RGB space. Visually from figure (Fig. 4d), we can see that brightness is the main feature distinguishing each kernel from the touching areas (where the kernel edges should be). Therefore the colour space was converted to extract the brightness feature. Figure 6 shows the typical maize fruit images to be enhanced. Figure 6 shows the Lab color images expressed by BGR model.

4 $$ \left\{ {\begin{array}{ll} {L = 0.2126R + 0.7152G + 0.0722B} \\ {a = 1.4749\left( {0.2213R - 0.339G + 0.1177B} \right) + 128} \\ {b = 0.6245\left( {0.1949R + 0.6057G - 0.8006B} \right) + 128} \\ \end{array} } \right. $$

Secondly, the CD Matrix was generated. The CD algorithm transforms the Lab colour space R to a new colour space R* composed of the stains used for staining the fruit. The transformation between R and R* is defined by Lambert-Beers law as the following Eq. (5):

5 $$ R = exp\left( { - \varvec{C}R^{*} } \right) $$

where $\varvec{C}$ is the optical density (OD) matrix composed of absorption factors *c* associated with stains, as Eq. (6):

6 $$ \varvec{C} = \begin{array}{*{20}c} {c_{l,1} } & {c_{a,1} } & {c_{b,1} } \\ {c_{l,2} } & {c_{a,2} } & {c_{b,2 } } \\ {c_{l,3} } & {c_{a,3} } & {c_{b,3} } \\ \end{array} $$

where $c_{l,1} ,c_{a,1}$ and $c_{b,1}$ are the predefined, normalized *L, a* and *b* absorption factor for the first stain $c_{1}$. The transformation of Eq. (5) is Eq. (7),

7 $$ R^{*} = \varvec{D}R^{\prime} $$

8 $$ {\text{where}}\;{\mathbf{D}} = \varvec{C}^{ - 1} $$

9 $$ {\text{and}}\;R^{\prime} = - \ln R $$

Here, the optical density (OD) for three channels can be defined as R’. Each pure stain is characterized by a specific OD for the light in each channel (Lab), which can be represented by a 3 × 1 OD vector describing the stain in the Lab colour space. In the case of three stains, the new colour space can be described as a matrix of the form OD matrix, as Eq. (6). *D* is the CD matrix obtained by calculating the inverse of the OD matrix *C*. In our work, the OD matrix cannot be determined arbitrarily as maize fruit are not stained by specific dyes. A conclusion drawn by Ruifrok and Johnston is that stain amount distribution is not influenced by the combination of multiple stains [41].

If maize fruit image $I\left( {X, R} \right)$ is defined as a 2D set of pixels X with its colour space function R composed of L, a and b intensity, $I\left( {X,  R^{*} } \right)$ in R* colour space is calculated according to Eq. (7). This allows a distribution map of an individual stain in stain combination to be obtained.

In our experiment, the colour appearance of different maize varieties varied widely. We experimented to receive a better kernel enhancement result by testing 10 stain combinations and 80 images, i.e. 10 sample images for each of 8 maize varieties. Images of each variety were taken under various light conditions. For each image, we applied these 10 stain combinations and found the one with best separation performance. The evaluation of the performance was by going through the segmentation and recognition procedures to acquire the kernel counting results. These results were then compared with the ground truth. Those two procedures will be introduced in the following sections. Three stain combinations were chosen for their better performance of enhancing the edge information and they produced the best results for the 80 test images. Two were adapted from https://blog.bham.ac.uk/intellimic/g-landini-software/colour-deconvolution/, which is a colour deconvolution plugin for ImageJ and Fiji [41]. They are called methyl green and hematoxylin GL by the author. One was customized by ourselves and we call it “white”. The OD matrices are shown in Eqs. (10)–(12). In order to automatically choose the most suitable one from these three combinations for unseen images, we designed a method. It split the 80 test images into 3 groups on each a stain combination produced the best performance. The average L, a, b values of each group were calculated. So when an unseen image is input, we calculate its L, a, b values and choose the stain combination with the least Euclidean distance of Lab values.

10 $$  \begin{gathered}   \quad \quad \quad \quad \quad \quad \quad R\quad \quad \quad \quad G\quad \quad \quad \quad B \hfill \\   methylgreen\left[ {\begin{array}{*{20}l}    {0.98003\quad 0.144316\quad 0.133146} \hfill  \\   \end{array} } \right] \hfill \\  \end{gathered}   $$

11 $$  \begin{gathered}   \qquad \quad \quad \quad \quad \quad \quad R\quad \quad \qquad \quad G\quad \quad \quad \qquad B \hfill \\   hematoxylin\,GL\,\left[ {\begin{array}{*{20}l}    {0.644211\quad 0.716556\quad 0.266844} \hfill  \\   \end{array} } \right] \hfill \\  \end{gathered}  $$

12 $$ \begin{aligned} &\quad \qquad R  \quad G \quad  B \\ &white\,   \left[ {\begin{array}{ll} {0   }  \quad{           0   }  \quad{           0} \\ \end{array} } \right] \\ \end{aligned} $$

**Fig. 7:**
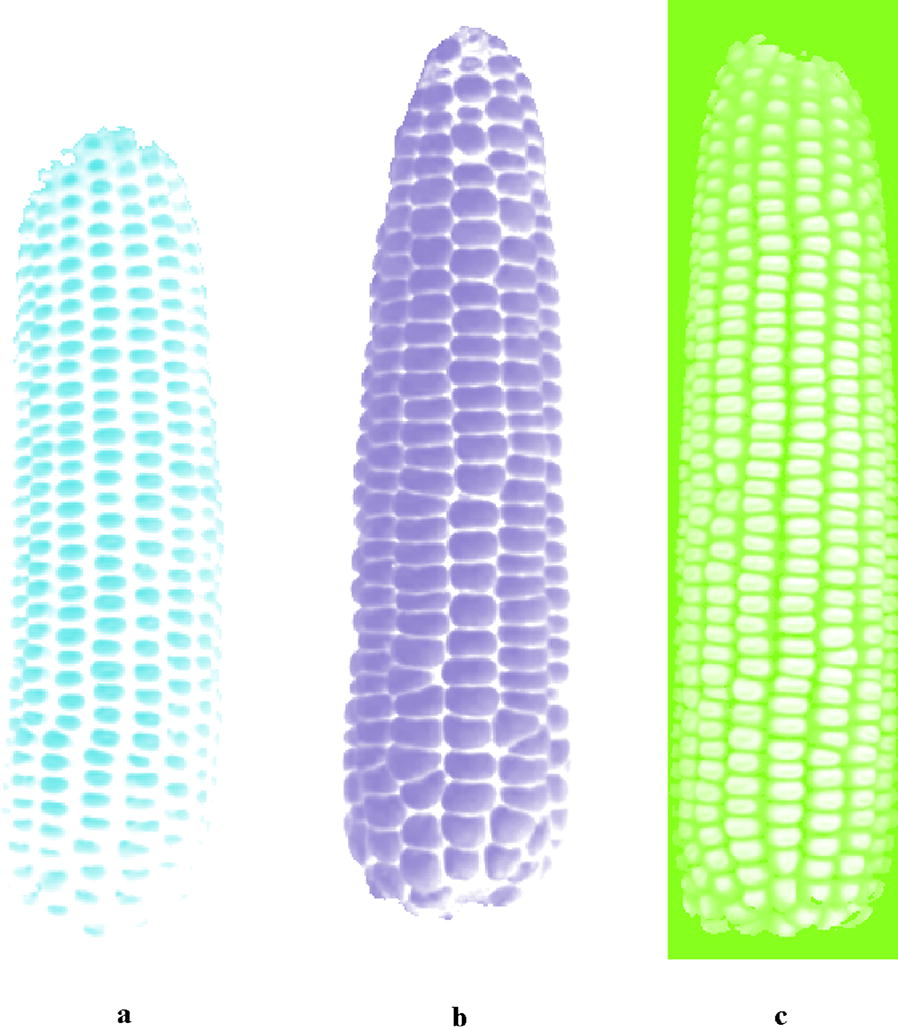
Edge enhancement maps: **a** Xianyu 688 “stained” by methyl green; **b** Jinnuo 685 “stained” by hematoxylin GL; **c** Fudan No. 3 “stained” by white

Xianyu 688, Jinnuo 685 and Fudan No.3 in Fig. 5 are three typical varieties for each stain combination methyl green, hematoxylin GL and white. The edge enhancement maps corresponding to Fig. 6, processed by CD algorithm, are shown in Fig. 7. Figure 7 shows a clearer definition of the kernel edge, demonstrating that CD algorithm is propitious to edge enhancement.

#### Kernel segmentation

**Fig. 8:**
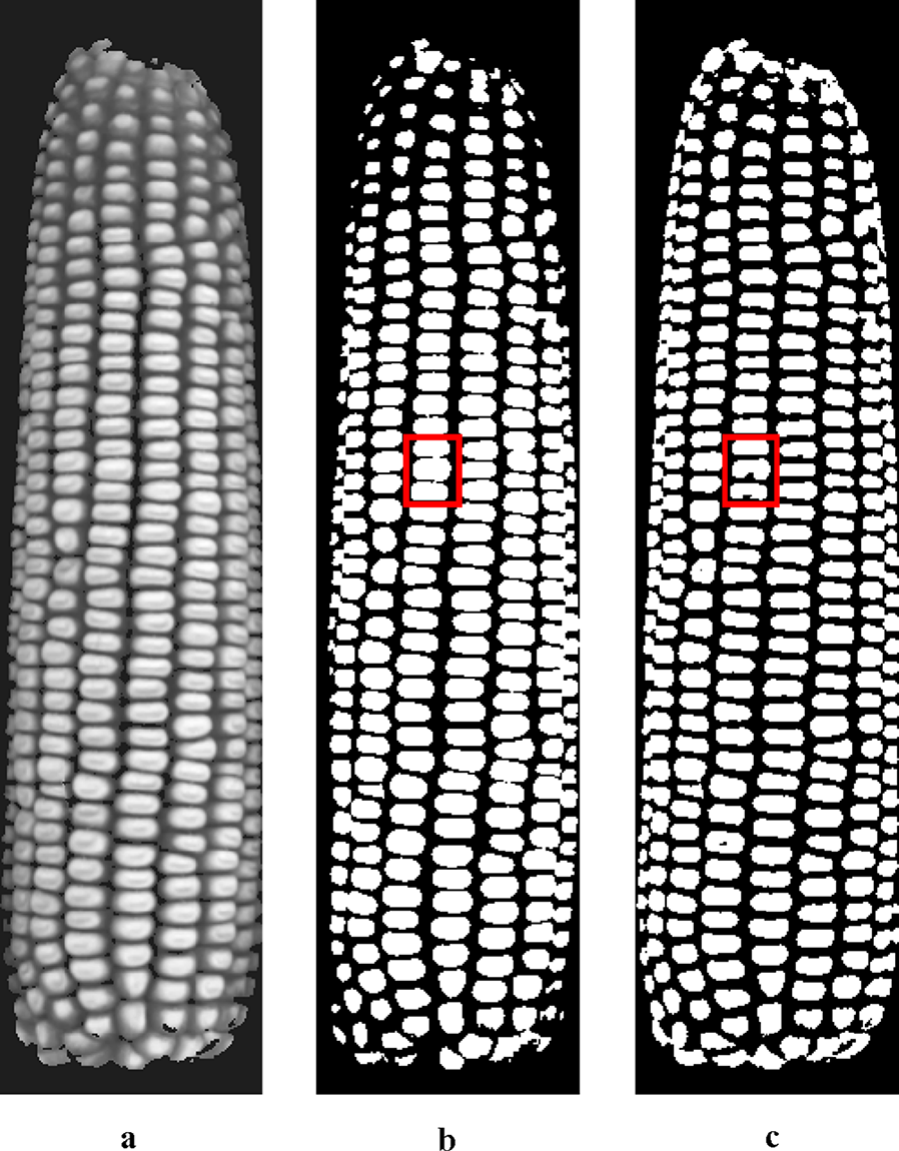
Gray image and binary image after segmentation: **a** gray image; **b** Otsu threshold segmentation result; **c** adaptive threshold segmentation result

The gray image extracted from Fig. 7c, taking R channel from the processed image, is shown in Fig. 8a. Due to uneven distribution of light on the kernel, gray intensities are not continuously uniform in the global range. Fixed threshold segmentation would result in over-segmentation or under-segmentation. Figure 8b shows the Otsu (Maximum Between-Class Variance) threshold segmentation image in which touching kernels in red rectangular box are under-segmented. An adaptive threshold method for compensation of the lighting or reflecting unevenness was considered for separating the kernels [39]. Every pixel *c(i, j)* in the grayscale image has one threshold *M(i, j)* which is the mean gray value in neighborhood block centered on *c(i, j)*, and then pixel *c(i, j)* with gray value larger than M is set to 255 or otherwise to 0. The size of neighborhood block *blocksize* is set to the average length of kernels minimum bounding rectangle (MBR), experimentally. Too small a *blocksize* results in more noise, while an oversized one may lose edge information. Figure 8c shows the adaptive threshold segmentation result. Compared to Fig. 8b, under-segmented and over-segmented kernels are greatly reduced in Fig. 8c, which demonstrated that the adaptive threshold method is suitable for kernel segmentation.

#### Kernels recognition

**Fig. 9:**
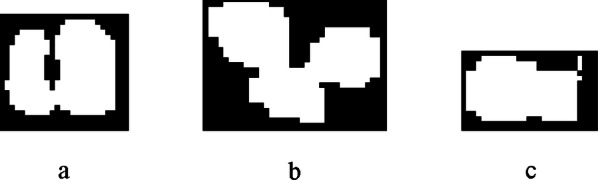
Binary touching kernels images: **a** corner-to-corner touching kernels; **b** edge-to-corner touching kernels; **c** edge-to-edge touching kernels

Figure 8c shows that after segmentation, there are still touching kernels on both sides of the maize fruit owing to lighting factors and kernel occlusions. The adhesion type includes not only corner-to-corner and edge-to-corner touching but also edge-to-edge touching, which are shown in Fig. 9.

For such a complicated problem, a local maximum detection method based on Gaussian filter was applied to recognize kernels.

**Fig. 10:**
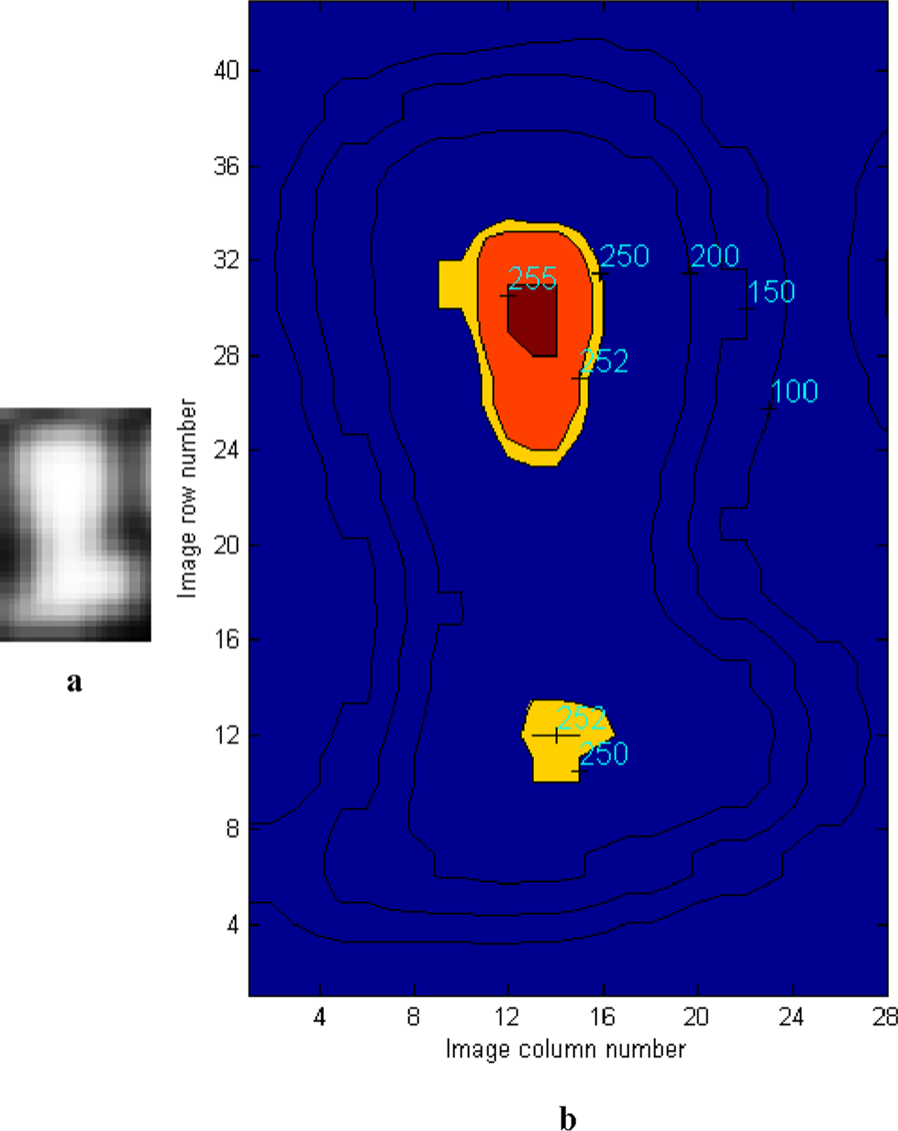
Gaussian filtering result of edge-to-edge touching kernels image: **a** result image; **b** contour map

Step 1: Smoothing the kernel binary image with Gaussian filter. This step aims to enhance the intensity at the kernel center which is the maximum point to be detected. Thus, the closer to the kernel center, the greater the intensity level. Figure 10a shows the smoothing result of the edge-to-edge touching kernels image. Figure 10b illustrates that the maxima points are distributed at the center of a kernel after Gaussian filtering. The size of the Gaussian filter window ${\text{N*N}}$ was determined to be the average width of seed MBR (minimum bounding rectangle).

Step 2: Defining a local block with a rectangular box which moves line by line in the image and finds the maxima points as initial kernel recognition points in the local block. The size of the local detection block ${\text{L*L}}$ was determined to be the average width of kernel MBR, which helps to find the recognition point corresponding to the kernel.

**Fig. 11:**
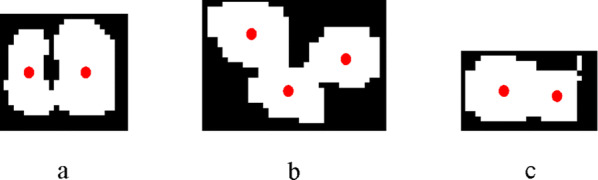
Recognition effect: **a** corner-to-corner touching kernels; **b** edge-to-corner touching kernels; **c** edge-to-edge touching kernels

Step 3: Eliminating spurious kernel recognition points. Multiple maxima points were obtained corresponding to the same kernel, and the chosen recognition point needs to be positioned to the centroid location of the local block for accurate counting. The recognition results for images in Fig. 9 are shown in Fig. 11.

## Results

### Recognition performance

A total eight maize ear varieties were used to develop the proposed automatic recognition algorithm and evaluate its performance. Two illumination conditions were used to test the robustness about imaging environments of our method. The ground truth is manual counting of kernel number in a maize ear side view image. The performance of our proposed algorithm was also compared with that of Hough Circle detection algorithm that has been widely applied to the detection of objects that contain circular feature to some extent such as apples, disease spots and arcs in agriculture and industry [34, 35]. The Hough Circle detection has the following advantages: 1. It is suitable for positioning multi-circle with short diameter. 2. The circular objects that obscured by others or the irregular short arcs can be detected. The algorithm was therefore considered to be suitable for recognizing touching and occluded kernels on maize ear. The test examples for eight varieties are listed below.

**Fig. 12:**
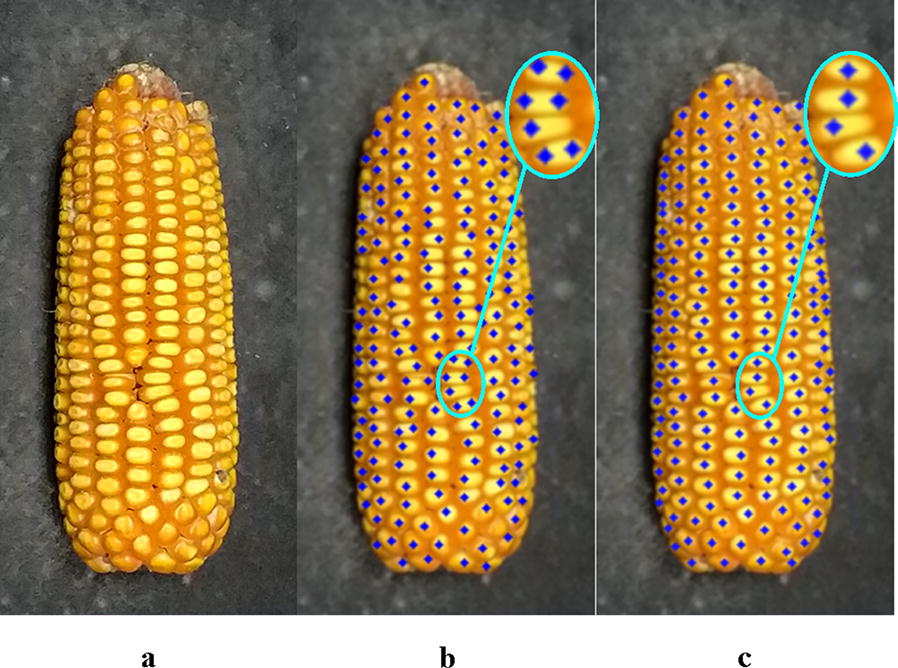
Recognition results of test sample Zhengdan 958: **a** original image of maize ear; **b** recognition effect based on Hough Circle detection algorithm; **c** recognition effect based on the proposed algorithm

**Fig. 13:**
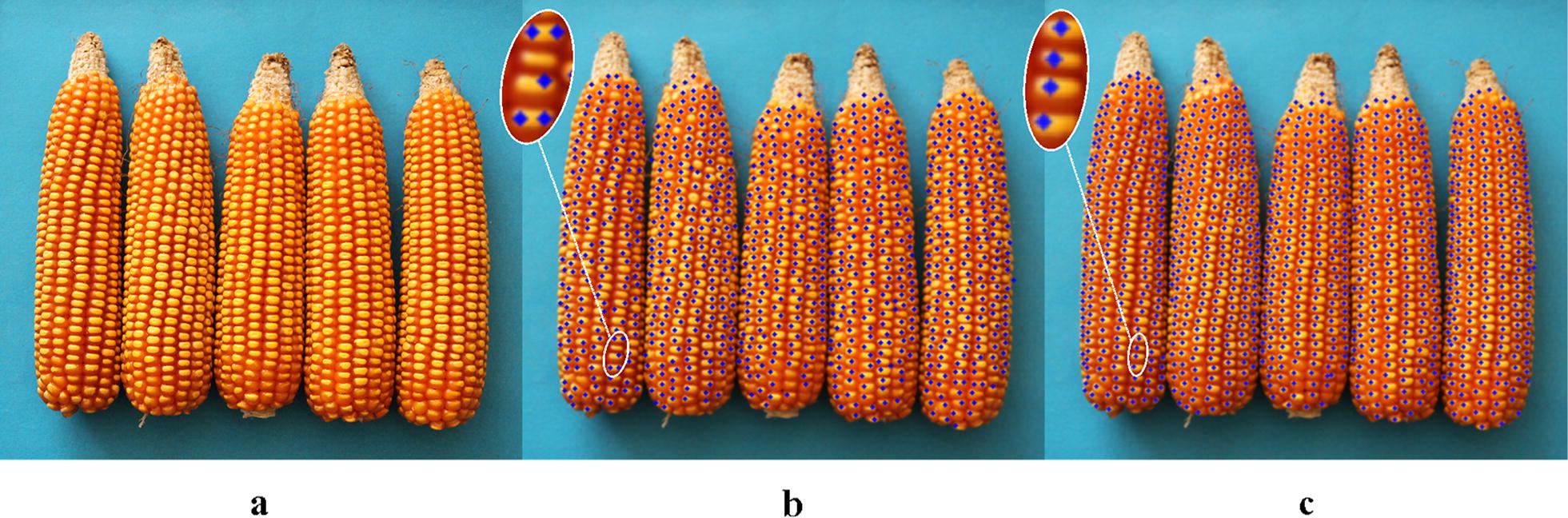
Recognition results of test sample Xianyu688: **a** original image of maize ear; **b** recognition effect based on Hough Circle detection algorithm; **c** recognition effect based on the proposed algorithm

**Fig. 14:**
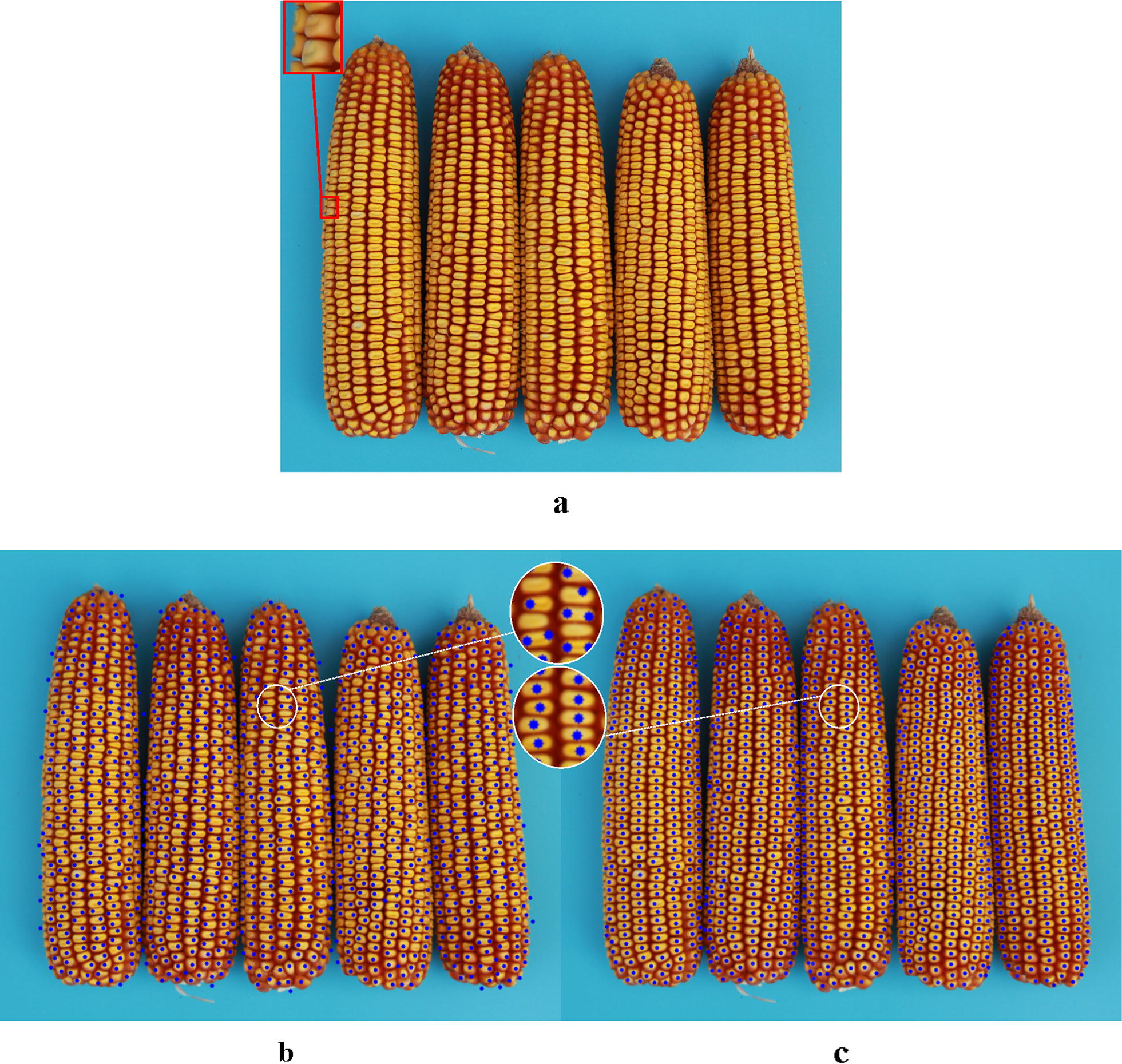
Recognition results of test sample Fudan No. 3: **a** original image of maize ear: kernel occlusion on the ear side is shown in the red box; **b** recognition effect based on Hough Circle detection algorithm; **c** recognition effect based on the proposed algorithm

**Fig. 15:**
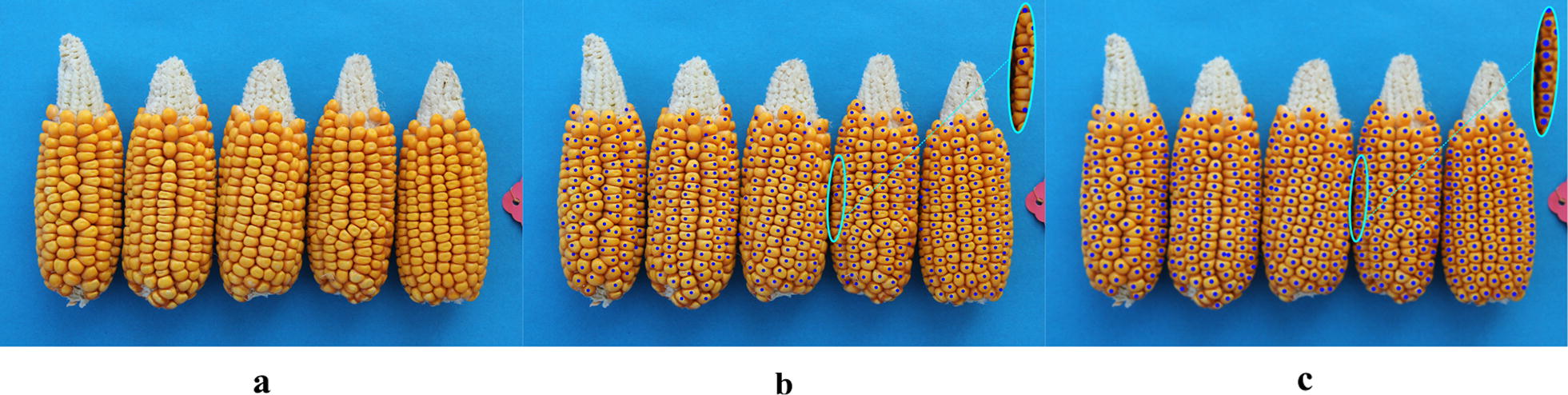
Recognition results of test sample Xianyu 335: **a** original image of maize ear; **b** recognition effect based on Hough Circle detection algorithm; **c** recognition effect based on the proposed algorithm

**Fig. 16:**
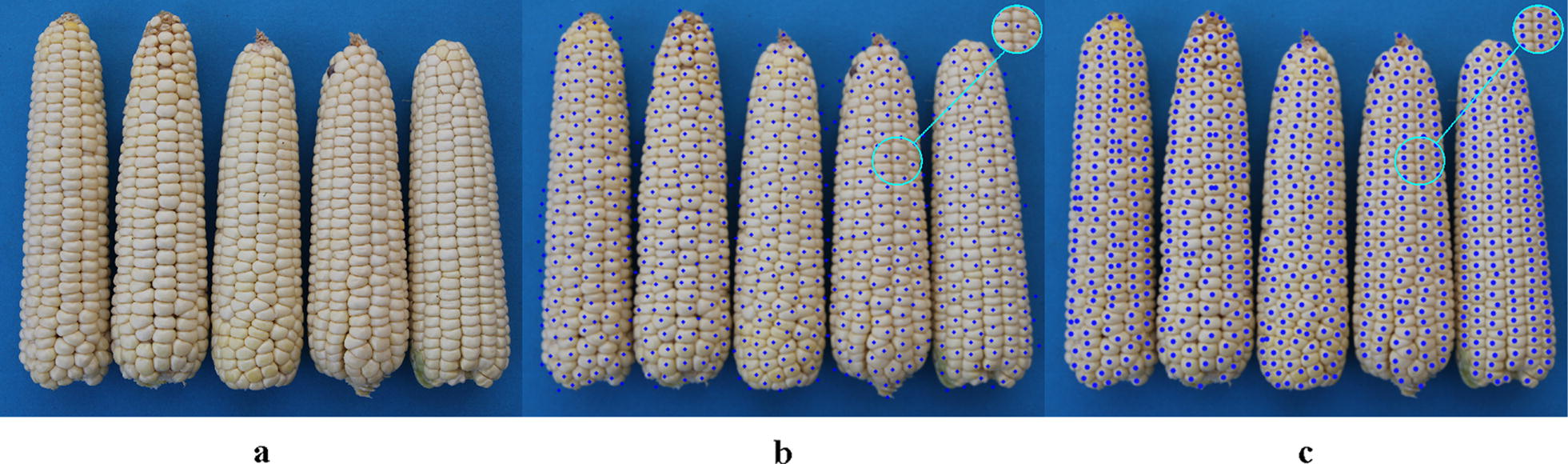
Recognition results of test sample Jinnuo 685: **a** original image of maize ear; **b** recognition effect based on Hough Circle detection algorithm; **c** recognition effect based on the proposed algorithm

**Fig. 17:**
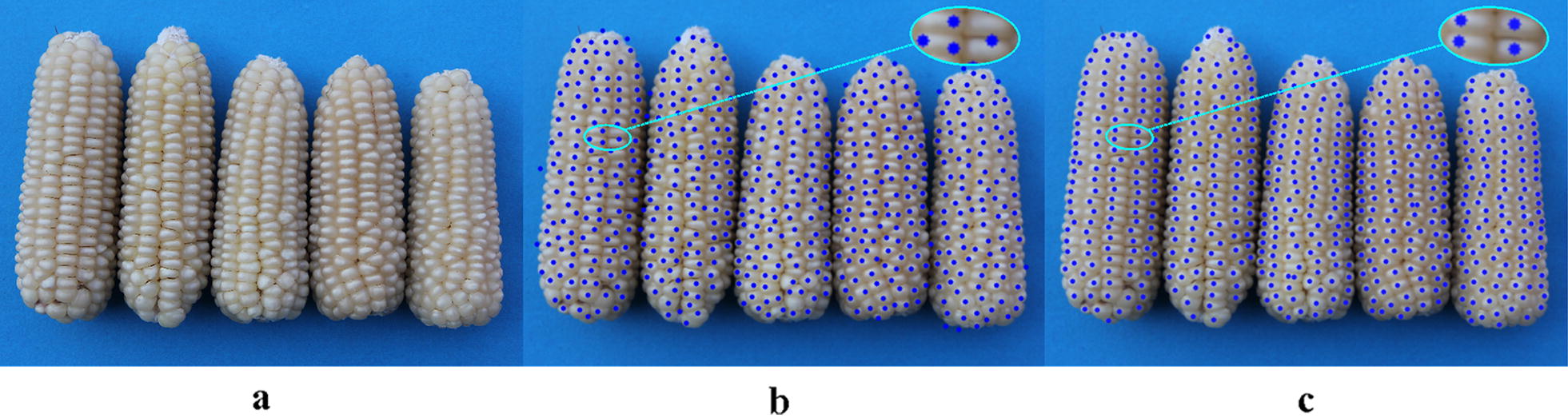
Recognition results of test sample Jingkenuo 2016: **a** original image of maize ear; **b** recognition effect based on Hough Circle detection algorithm; **c** recognition effect based on the proposed algorithm

**Fig. 18:**
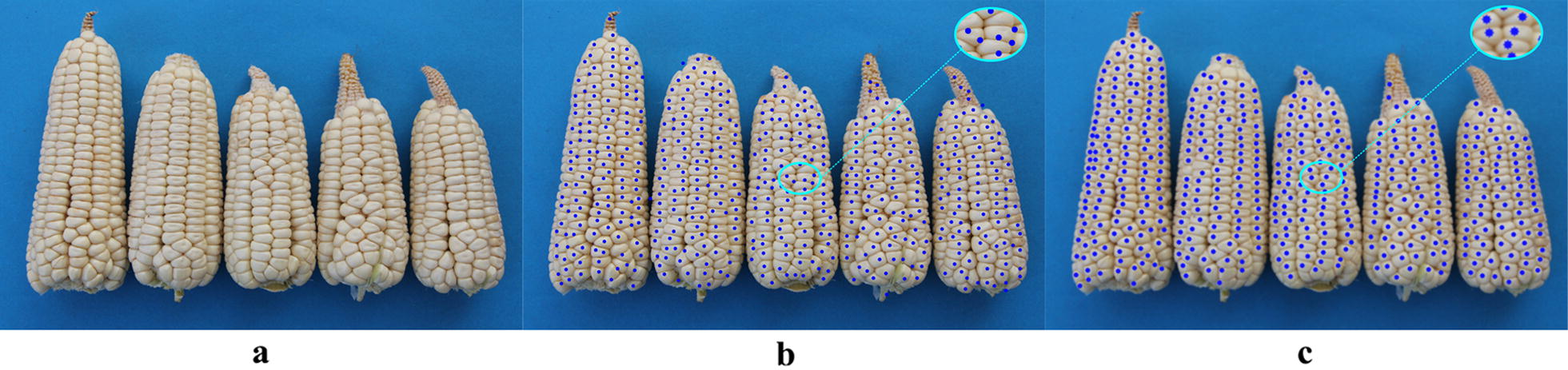
Recognition results of test sample Beibainuo No. 10: **a** original image of maize ear; **b** recognition effect based on Hough Circle detection algorithm; **c** recognition effect based on the proposed algorithm

**Fig. 19:**
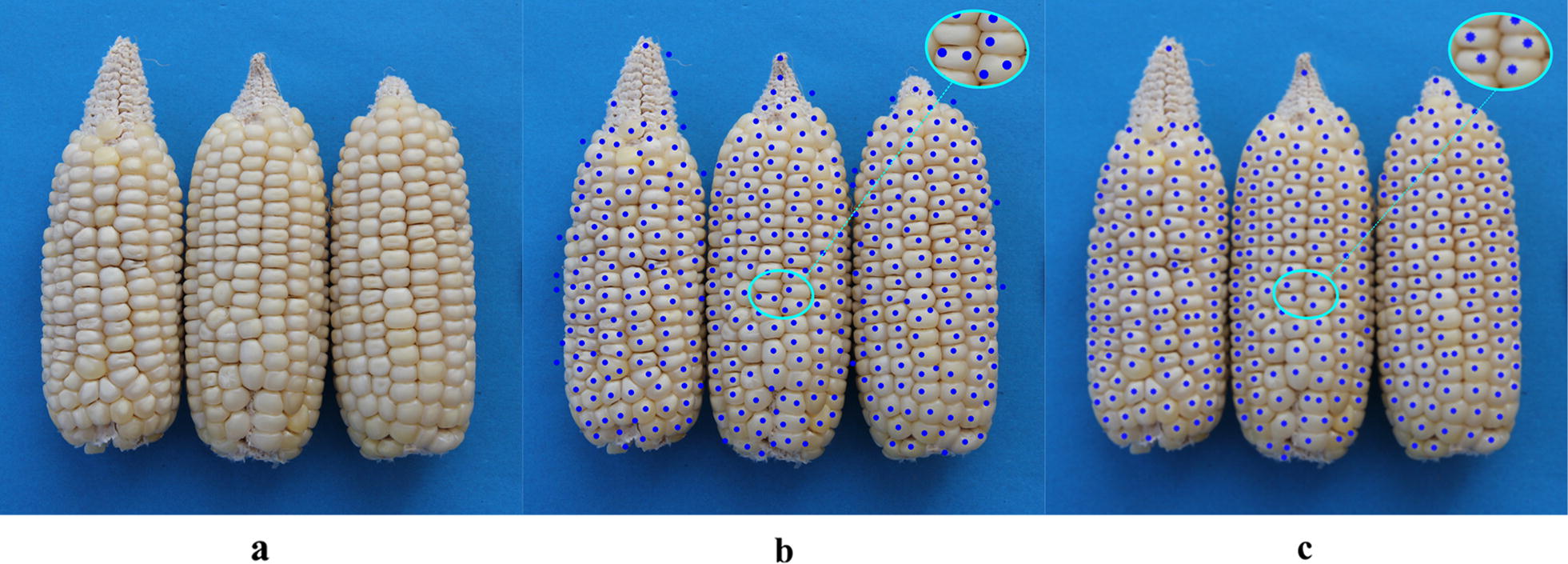
Recognition results of test sample Zhengbai No. 1: **a** original image of maize ear; **b** recognition effect based on Hough Circle detection algorithm; **c** recognition effect based on the proposed algorithm

Comparisons between Hough Circle detection and the proposed algorithm used eight varieties, i.e. Zhengdan 958, Xianyu 688, Fudan No. 3, Xianyu 335, Jinnuo 685, Jingkenuo 2016, Beibainuo No. 10 and Zhengbai No. 1 (Figs. 12, 13, 14, 15, 16, 17, 18 and 19). As shown in Figs. 12a, 13, 14, 15, 16, 17, 18 and 19a, all kernels on the maize ear are interconnected to each other in the original images, some are even occluded, and their shapes vary widely. Figures 12b, 13, 14, 15, 16, 17, 18 and 19b show the results by Hough Circle detection algorithm. Most kernels were well recognized. However, a kernel was either unrecognized or recognized as two when its contour roundness is low (shown in the partial enlarged view), which reduced the counting accuracy. Figures 12c, 13, 14, 15, 16, 17, 18 and 19c show the results by our proposed algorithm, where the cases not recognized correctly by Hough Circle detection were now recognized. Particularly, the proposed algorithm could recognize kernels with diverse touching types. A few areas belonging to bald tip were both recognized as kernels by two algorithms (Figs. 18, 19), but Hough Circle detection produced more false positives. Indoor LED diffuse and outdoor natural diffuse illumination were respectively applied in the test. The Zhengdan958 image (Fig. 12) was acquired under indoor LED diffuse which has a narrow spectral range, so it is a little more warm yellow. Highlights appeared in the maize area near the LED tube and shadows appeared in the maize area occluded by the camera. Different from other varieties, the Zhengdan958 images were collected at a farmhouse in Suzhou City, Anhui Province, where there is no blue background, we replaced it with a black background. But that does not affect the background segmentation process. In contrast, other samples (Figs. 13, 14, 15, 16, 17, 18 and 19) were acquired under outdoor natural light around 10 a.m. which has a balanced spectrum. Light is evenly distributed in maize ear area. The results (Figs. 12c, 13, 14, 15, 16, 17, 18 and 19c) did not show clear influences caused by illumination conditions.

**Fig. 20:**
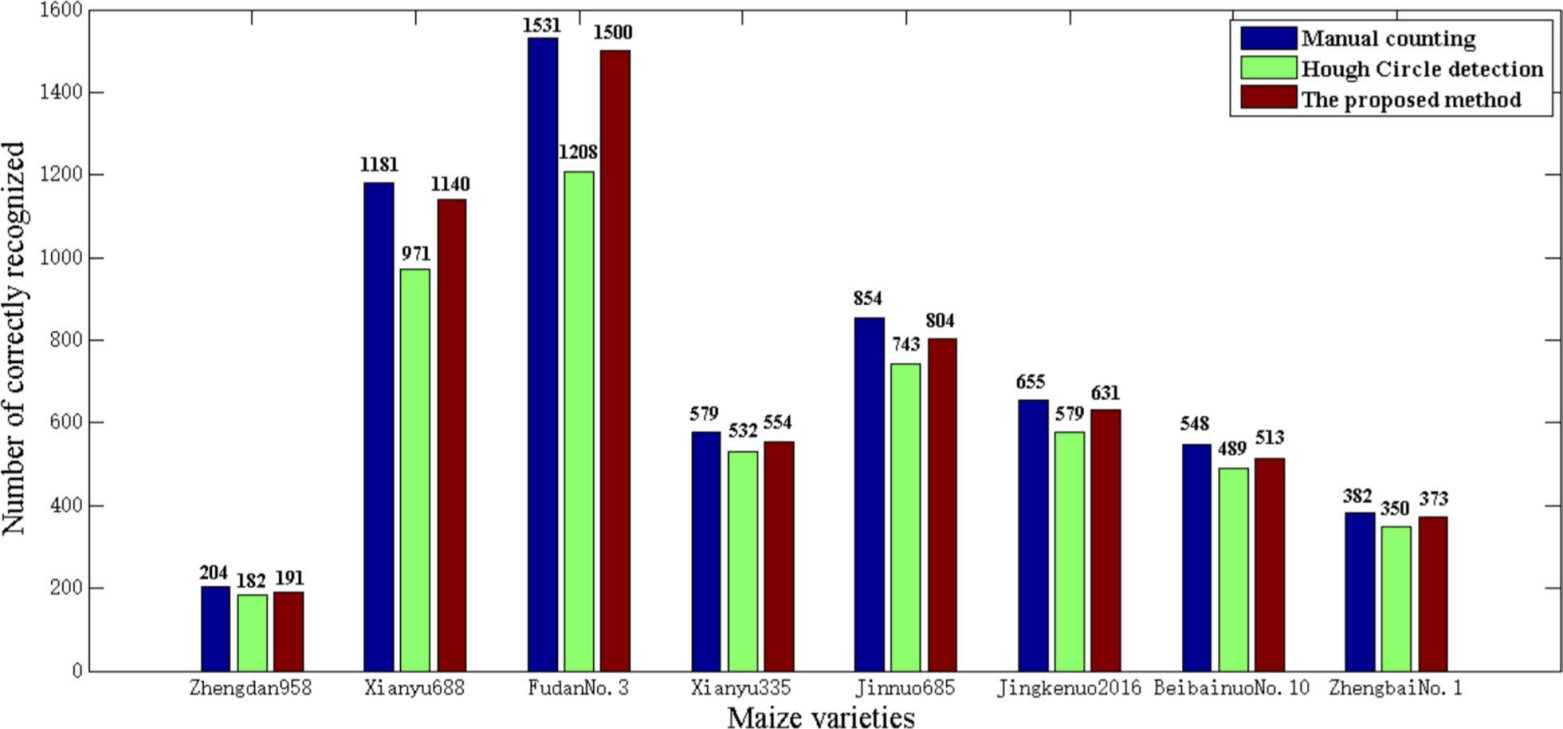
Counting results of two recognition algorithms for eight different varieties

Then the proposed algorithm was evaluated quantitatively. The results are shown in Table 1. The correct recognition rate of the proposed method was more than 93.6%. In contrast, Hough Circle detection is less than 90% with the lowest rate being 78.9%. Compared with Hough Circle detection algorithm, the false positive and false negative numbers of the proposed method were greatly reduced. Figure 20 shows how accurate our proposed algorithm is. Table 2 shows the results in batch testing. The average correct rate is the average of individual correct recognition rates on each maize ear, which shows good consistency between the batch test results and the single test results. The above results indicate that the proposed algorithm has superior performance. It is more accurate compared with traditional methods, such as the Hough Circle detection algorithm, for maize kernel recognition in ear image. The results also show that the algorithm is robust under different illumination conditions. The average processing time by the proposed algorithm on a single ear is 0.64 s. Therefore, the proposed method is efficient and accurate for the automatic recognition and counting of maize kernels.

**Table 1:** Comparisons of counting accuracy of two recognition algorithms for eight different varieties

Maize varieties	Number of kernels	Hough circle detection algorithm				Proposed algorithm			
		Number of correctly recognized	False-positive number	False-negative number	Correct rate (%)	Number of correctly recognized	False-positive number	False-negative number	Correct rate (%)
Zhengdan 958	204	182	6	22	89.2	191	0	13	93.6
Xianyu 688	1181	971	9	210	82.2	1140	2	41	96.5
Fudan No. 3	1531	1208	51	223	78.9	1500	30	31	97.9
Xianyu 335	579	532	19	47	91.9	554	3	25	95.7
Jinnuo 685	854	743	33	111	87.0	804	15	50	94.1
Jingkenuo 2016	655	579	38	76	88.4	631	2	24	96.3
Beibainuo No. 0	548	489	43	59	89.2	513	8	35	93.6
Zhengbai No. 1	382	350	49	32	91.6	373	13	9	97.6

**Table 2:** Counting accuracy results in batch tests

Maize varieties	Number of ears	Hough circle detection algorithm	Proposed algorithm
		Average correct rate (%)	Average correct rate (%)
Zhengdan 958	80	87.6	94.3
Xianyu 688	160	83.9	96.2
Fudan No. 3	160	79.2	97.1
Xianyu 335	160	90.7	95.2
Jinnuo 685	160	88.5	95.2
Jingkenuo 2016	160	87..8	96.1
Beibainuo No. 10	160	88.9	94.1
Zhengbai No. 1	160	92.1	97.8

### Segmentation by morphological algorithm

**Fig. 21:**
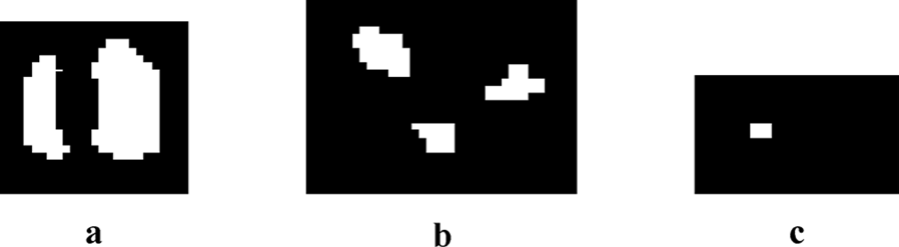
Corrosion result image: **a** corner-to-corner touching kernels; **b** edge-to-corner touching kernels; **c** edge-to-edge touching kernels

Figure 21 shows the results of repeated morphological corrosions on three touching types (as derived from Fig. 9) until the kernels are separated. Corner-to-corner and edge-to-corner touching kernels can be separated, but edge-to-edge touching kernels cannot be separated until the smaller one disappeared, which results in counting error.

### Segmentation of touching kernels

**Fig. 22:**
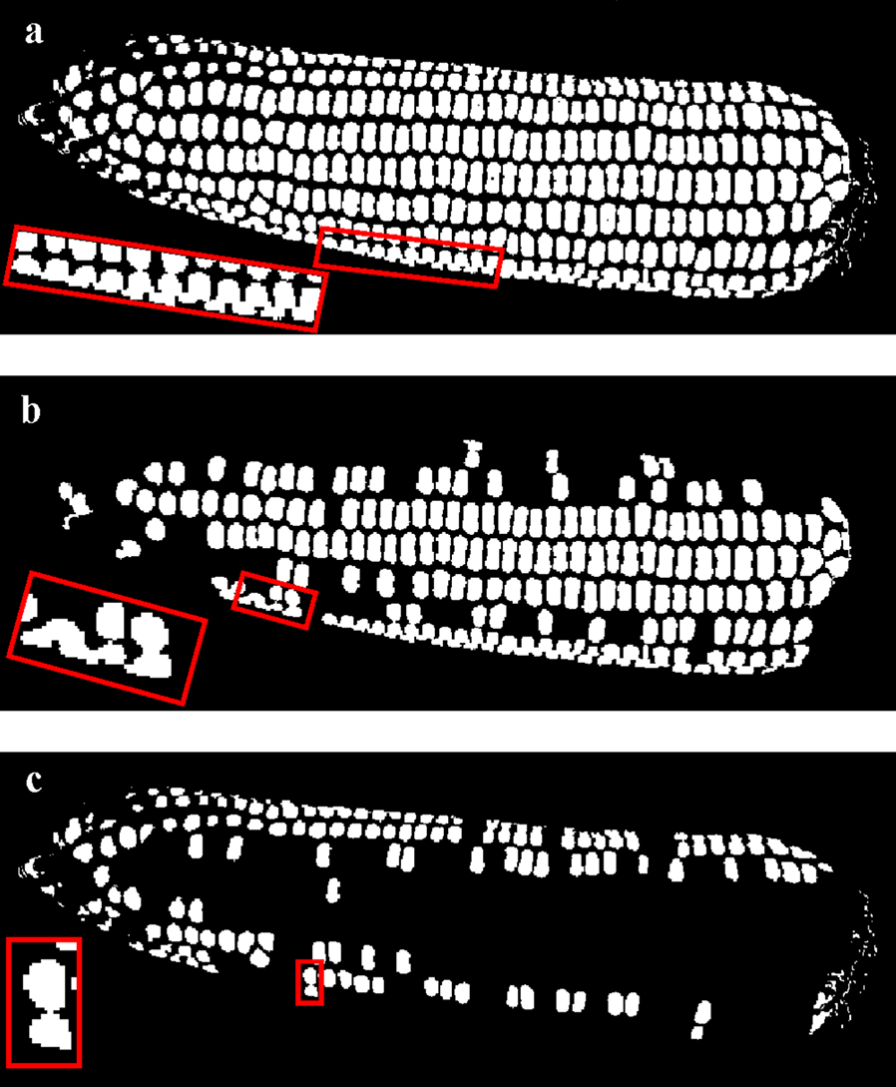
Segmentation result image by area threshold: **a** binary image after segmentation; **b** kernels with area greater than threshold; **c** kernels with area less than threshold

The kernels were separated into two categories according to individual kernel area threshold which was set to the average of the areas. Figure 22b, c respectively demonstrated kernels with their area greater and less than the average area, in which there are both individual and touching kernels (shown in the red box). Therefore, the segmentation method based on area threshold cannot be used to judge whether the kernel is touching. Obviously, our method could deal with these states.

## Discussion

Counting kernel number of a maize ear through image processing is challenging but of significance for maize breeding. As shown in Figs. 12a, 13, 14, 15, 16, 17, 18 and 19a, the fuzzy touching area between kernels, with narrow colour gradients results in almost all kernels on the maize ear touching each other. Some kernels are even occluded, and their shapes vary widely. As can be seen from segmentation results using morphological algorithm, morphology and morphology-based seed watershed methods, these may not be suitable for segmenting the kernels in maize ear image. In contrast, the accuracy of our proposed method is suitable for practical applications in maize kernels recognition, as it exceeded 93% for eight typical ear varieties. This demonstrates that the method has good stability for multiple maize varieties. Compared with Hough Circle detection algorithm, the proposed method is able to take account of irregular kernel shapes. Moreover, the method is efficient: processing $700 \times 2300$ pixel image takes about 0.64 s on a computer, with Intel(R) Core(TM) i5-4210U CPU @ 1.70 GHz 2.40 GHz, 12G (RAM) but without using a GPU. That makes real-time processing using low cost hardware feasible. According to the results by area threshold, whether increasing or decreasing the area threshold, the touching and individual kernel cannot be correctly classified. It is because of the varying kernel sizes. Nevertheless, the importance of our method is two-fold: Firstly, it can recognize touching kernels of irregular shapes in the same maize ear. Secondly, there is no need to distinguish touching and individual kernels in recognition.

We can see that there are a few false recognition points on the bald tip of maize only for two varieties Figs. 18, 19. This is because that some areas on the bald tip would not be removed in the step background separation due to its redundant color intensity and were processed as fruit in subsequent steps. It has a slight impact on counting precision and can be ignored. Due to one-sided imaging of the ear, it is inevitable that occlusion will arise on both sides of the maize ear, as shown in the red box of Fig. 14a. In addition, because of weakened illumination on both sides, the occluded and small kernel is much smaller than other kernels. So the size of the touching area approximates that of a single kernel, which leads to a negative recognition error as the occluded kernels are not recognized by the proposed algorithm and affects the accuracy of automatic counting. Thus, further research is needed to solve the problem of the recognition of occluded maize kernel. We hope to avoid occluded kernels by improving image acquisition methods, such as panoramas. Due to the imaging angle, the kernel size varies greatly that the block size is relatively small for some kernels and the kernel is recognized as two. Thus, further work is needed to reduce false-positive number by distortion correction. In experiment of verifying the robustness of the algorithm to lighting, we did not capture all samples under two illuminations. The work will be improved in later research to make the results more convincing.

## Conclusion

This paper proposes a high-efficiency and low-cost approach to recognize and count the kernels on maize ear. Due to its accurate counting, fast speed and stable operation, the kernel recognition algorithm can be used as an alternative to the traditional manual counting especially in a high throughput manner. Moreover, this algorithm is applicable to counting multiple varieties of maize kernels. In our experiment, two different lighting conditions are considered, where the algorithm performs well. Nevertheless, the accuracy of this method is affected when the kernels on both sides of the ear are occluded and the image geometry distortion is large. This method also provides a reference for the counting of other crop kernels, which will be helpful for breeding programs.

## Data Availability

All data generated or analysed during this study are included in this published article.
